# DoriC 10.0: an updated database of replication origins in prokaryotic genomes including chromosomes and plasmids

**DOI:** 10.1093/nar/gky1014

**Published:** 2018-10-26

**Authors:** Hao Luo, Feng Gao

**Affiliations:** 1 Department of Physics, School of Science, Tianjin University, Tianjin 300072, China; 2 Key Laboratory of Systems Bioengineering (Ministry of Education), Tianjin University, Tianjin 300072, China; 3 SynBio Research Platform, Collaborative Innovation Center of Chemical Science and Engineering (Tianjin), Tianjin 300072, China

## Abstract

DoriC, a database of replication origins, was initially created to present the bacterial *oriC*s predicted by Ori-Finder or determined by experiments in 2007. DoriC 5.0, an updated database of *oriC* regions in both bacterial and archaeal genomes, was published in the 2013 Nucleic Acids Research database issue. Now, the latest release DoriC 10, a large-scale update of replication origins in prokaryotic genomes including chromosomes and plasmids, has been presented with a completely redesigned user interface, which is freely available at http://tubic.org/doric/ and http://tubic.tju.edu.cn/doric/. In the current release, the database of DoriC has made significant improvements compared with version 5.0 as follows: (i) inclusion of *oriC*s on more bacterial chromosomes increased from 1633 to 7580; (ii) inclusion of *oriC*s on more archaeal chromosomes increased from 86 to 226; (iii) inclusion of 1209 plasmid replication origins retrieved from NCBI annotations or predicted by *in silico* analysis; (iv) inclusion of more replication origin elements on bacterial chromosomes including DnaA-trio motifs. Now, DoriC becomes the most complete and scalable database of replication origins in prokaryotic genomes, and facilitates the studies in large-scale *oriC* data mining, strand-biased analyses and replication origin predictions.

## INTRODUCTION

In all living organisms, DNA replication is regulated precisely at the assembly stage of the replication machinery (1). Replication origins are the particular genomic loci, where double-stranded DNA unwinds to form single-stranded DNA templates to initiate synthesis of new strands. In most bacteria, the replication origin (*oriC*) contains several DnaA box motifs recognized by the principal initiator protein DnaA, and a region with high AT content, namely, the DNA unwinding element (DUE), where single-stranded DNA is also recognized by DnaA (2–5). Similarly, AT-rich DNA unwinding element is also found to be essential in archaeal replication origin, which is flanked by the origin recognition boxes (ORBs) that serve as binding sites for origin recognition proteins (6,7). In a large number of plasmids, the origin of vegetative replication (*oriV*) often consists of direct repeats or iteron DNA sequences, which interact with Rep proteins to form the initial complex during the process of replication initiation (8). There is also an AT-rich region near the location of iterons in *oriV*, which serves as the DNA unwinding element (9).

It is interesting that the replication origin is usually next to the replication-related genes, such as *dnaA, orc*1/*cdc*6 and *rep* genes. The similar structures of prokaryotic replication origins on chromosomes and plasmids provide the opportunity to design algorithms for origin prediction based on the same framework. Initially, Ori-Finder was developed to identify the *oriC* regions on bacterial chromosomes (10).

As a separate domain in the three-domain system, most archaea exist in various extreme environments on earth, and the particular habits make it difficult to identify their replication origins by experimental methods (11). Therefore, the web-based tool Ori-Finder 2 was developed to predict the *oriC* regions in archaeal genomes *in silico*, and the predicted results can be helpful in the identification of archaeal origins in the laboratory (12).

Plasmids are extrachromosomal auto-replicating genetic elements, which are widespread in bacteria, archaea, yeast and some higher eukaryotic cells (8). Plasmids often carry genes that bring some special features to the host cells, such as antibiotic resistance and toxin–antitoxin system (13). Therefore, autonomous DNA replication of plasmids is critical for cell survival. The origin of vegetative replication is one of the most important elements in plasmid. Up to now, the location and characteristic of *oriV*s were well understood in a broad range of plasmids, such as RK2, F, P1, R6K and pPS10 plasmids (14–18). However, bioinformatics tools are urgently needed to identify *oriV*s automatically on plenty of sequenced plasmids.

The predictions of Ori-Finder system were organized into an online database to facilitate the related research on replication origins (19–21). In 2007, DoriC, a database of *oriC* regions, was first publicly available to present the bacterial *oriC*s, and in 2013, DoriC 5.0 included replication origins in both bacterial and archaeal genomes (22,23). In the past six years, the rapid progress in next-generation sequencing technologies and the accumulation of sequenced genomes from various microbial genome projects have promoted the expansion of DoriC, and this expanded database is presented here as DoriC 10.0, which includes the replication origins of plasmids for the first time. DoriC database and Ori-Finder system ensure a better understanding of the structure and function of replication origins, and provide new insights into the regulatory mechanisms of the initiation step in DNA replication. So far, many of the predictions stored in DoriC are now verified in the laboratory, and more applications based on DoriC database and Ori-Finder system in the past years have been reviewed in our recent article (24).

## DATABASE UPDATES

**Figure 1.**
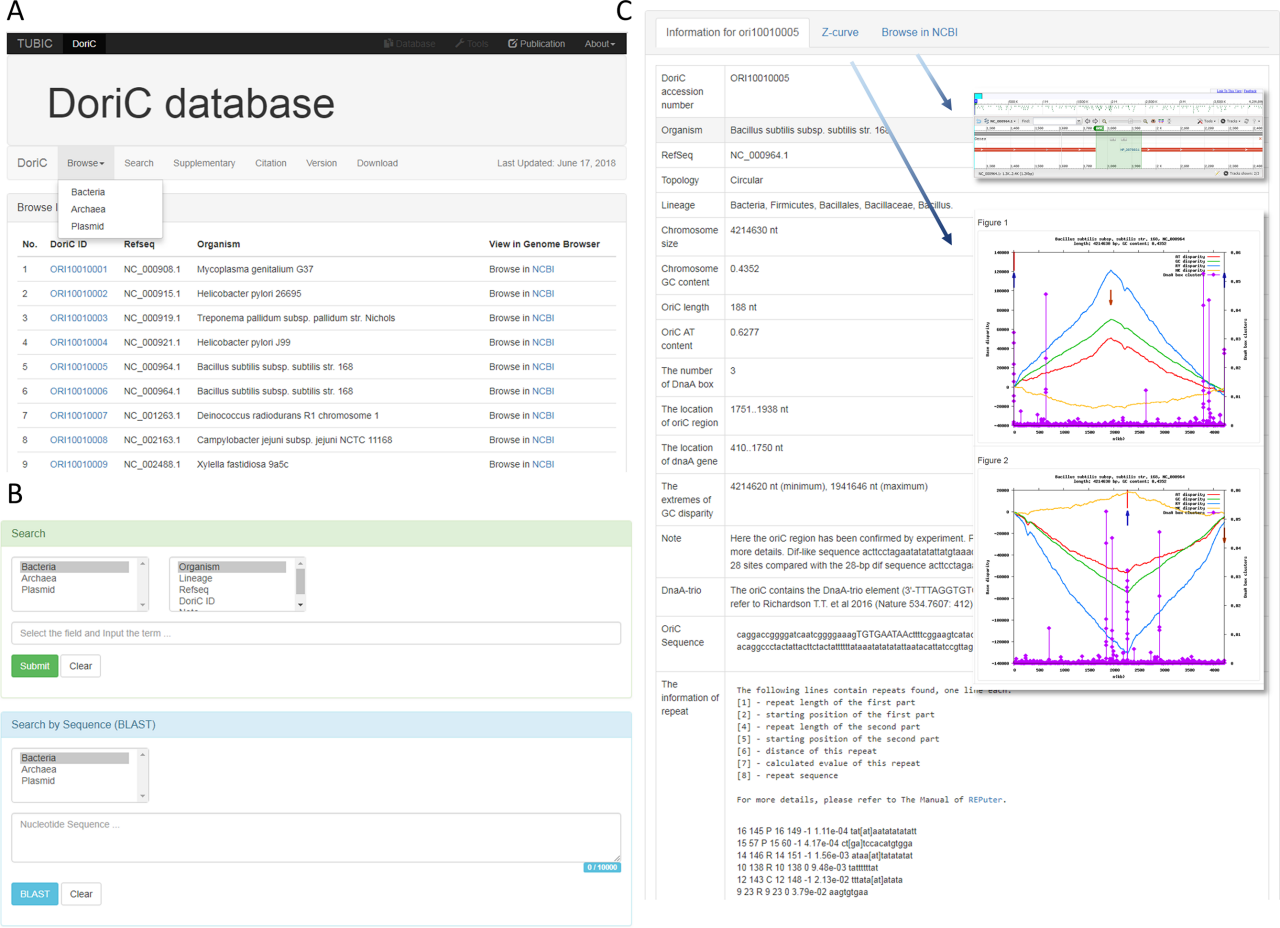
Snapshot of the redesigned user interface of DoriC. (**A**) Database browse interface for accessing the whole DoriC records of bacteria, archaea or plasmids. (**B**) Database search interface including record query and BLAST search. (**C**) A representative view of the record in DoriC displaying the information of replication origin, such as the location, sequence, DnaA-trio element and repeats. It can switch over to the Z-curve figures or the NCBI genome browser by Tabs.

In the current release, the content of DoriC is significantly improved compared with version 5.0 as follows: (i) the *oriC*s on bacterial chromosomes have increased fourfold from 1633 to 7580; (ii) the *oriC*s on more archaeal chromosomes have increased from 86 to 226; (iii) 1209 plasmid replication origins are presented for the first time, including 348 annotated origins retrieved from NCBI records and 861 predicted origins by a modified Ori-Finder system; (iv) more sequence elements in bacterial replication origins are incorporated, including DnaA-trio element, a new repeating trinucleotide motif important for origin function. DnaA-trios play a role in origin unwinding and DNA helicase loading, which are highly conserved throughout the bacterial kingdom by bioinformatics analysis with DoriC (20). The DnaA-trio-like sequence was searched in DoriC database, and then the information was supplemented to the corresponding *oriC* record. Furthermore, we redesigned the user interface of the database to make it more convenient and intuitive (Figure 1).

## DATABASE DESCRIPTION

### Replication origins on bacterial and archaeal chromosomes

Except those collected from scientific publications, the *oriC*s on bacterial chromosomes were predicted by Ori-Finder, which has also been used in the annotation of more than 120 newly sequenced bacteria in their genome reports. In general, the *oriC* predictions by Ori-Finder can be integrated into DoriC database directly. In some cases, manual curation based on the information from DoriC is required for the questionable predictions by Ori-Finder. For example, two potential *oriC*s were predicted in some *Gammaproteobacteria* genomes, but only the one next to the *gidA* gene was added to DoriC finally according to the enrichment of GATC motifs in *oriC* regions (21).

This large-scale update of DoriC provides new insights into the replication origins on bacterial chromosomes. For example, the *oriC* in cyanobacteria is usually adjacent to *dnaN* gene, and contains the species-specific DnaA boxes (TTTTCCACA) (25). However, the *oriC* with a cluster of DnaA boxes (TTTTCCACA) is located far from *dnaA* and *dnaN* genes in *Synechococcus lividus* PCC 6715. Besides that, adenine or thymine in some positions of the DnaA box motif ‘TTTTCCACA’ tends to offset to guanine or cytosine in some GC-rich genomes of cyanobacteria, such as *Cyanobium gracile* PCC 6307 (GC content: 0.6871).

The *oriC*s on archaeal chromosomes were predicted by Ori-Finder 2.0, which is mainly used to predict the replication origins adjacent to some replication-related genes, such as *cdc*6 gene (12). The ORB motifs used by Ori-Finder 2.0 were summarized based on the available experimentally determined *oriC*s stored in DoriC, and the understanding of the ORB motifs is still quite limited at the present stage. Therefore, some potential *orc*1/*cdc*6-adjacent replication origins without known ORB motifs were also included in DoriC if they are located around the extremes of disparity curves with significant repeats.

### Replication origins on plasmids

In this release, the *oriV*s on plasmids retrieved from NCBI annotations or predicted by *in silico* analysis have been collected into DoriC. For the *oriV*s retrieved from NCBI annotations, the intergenic regions, which have an overlap with the annotated replication origins according to NCBI records, are presented as potential *oriV*s in order to include as many repeat features as possible. The original location of *oriV* in the NCBI records was added as a note. The direct repeats or iterons DNA sequences in *oriV*s frequently conform to a consensus motif, but they are rarely identical in different plasmid sequences (26). In addition, the Rep proteins interact with inverted repeated (IR) sequences, causing transcriptional auto-repression (9,27). These repeat sequences were discovered in a great deal of *oriV*s according to NCBI annotations, and the information of repeats identified by REPuter pipeline was displayed in DoriC (28). Based on the characteristics of known replication origins on plasmids, *oriV*s were also predicted by a modified Ori-Finder system, and the intergenic regions, which are adjacent to *rep* genes with highly significant iterons DNA sequences and inverted repeats, were predicted as *oriV*s. The AT-rich regions in *oriV*s are usually followed by one or two DnaA-boxes (29), so the number of DnaA boxes identified in *oriV*s is also presented.

## CONCLUSION

With the significant advancements in sequencing technology, the number of sequenced microbial genomes has continued to increase dramatically, which presents both challenges and opportunities for the studies of replication origins. In this latest release, DoriC has stored over 9,928 records of prokaryotic chromosomal origins and included 1209 records of plasmid replication origins for the first time. As an essential database in microbial genomics, DoriC has been used in a large number of studies associated with the replication origins. In the past dozen years, the development of DoriC has promoted a better understanding of replication origins, and some records of replication origins in DoriC were confirmed by experiments. Despite significant progress in DoriC, there are still many issues needed to be addressed urgently. In the future, the database will be further improved with the continuous update by taking into account more scientific discoveries in the field of DNA replication and the predicted replication origins in draft genome sequences.
